# Revealing Alzheimer’s disease genes spectrum in the whole-genome by machine learning

**DOI:** 10.1186/s12883-017-1010-3

**Published:** 2018-01-10

**Authors:** Xiaoyan Huang, Hankui Liu, Xinming Li, Liping Guan, Jiankang Li, Laurent Christian Asker M. Tellier, Huanming Yang, Jian Wang, Jianguo Zhang

**Affiliations:** 1 BGI Education Center, University of Chinese Academy of Sciences, Shenzhen, 518083 China; 2 BGI-Shenzhen, Shenzhen, 518083 China; 3 China National GeneBank, BGI-Shenzhen, Shenzhen, 518120 China; 4 College of Life Science and Technology, Huazhong University of Science and Technology, Wuhan, China; 5 Department of Biology, Bioinformatics, University of Copenhagen, Copenhagen, Denmark; 6 James D. Watson Institute of Genome Sciences, Hangzhou, 310058 China; 7 Shenzhen Key Lab of Neurogenomics, BGI-Shenzhen, Shenzhen, 518120 China

**Keywords:** Alzheimer’s disease, Gene, Machine learning

## Abstract

**Background:**

Alzheimer’s disease (AD) is an important, progressive neurodegenerative disease, with a complex genetic architecture. A key goal of biomedical research is to seek out disease risk genes, and to elucidate the function of these risk genes in the development of disease. For this purpose, expanding the AD-associated gene set is necessary. In past research, the prediction methods for AD related genes has been limited in their exploration of the target genome regions. We here present a genome-wide method for AD candidate genes predictions.

**Methods:**

We present a machine learning approach (SVM), based upon integrating gene expression data with human brain-specific gene network data, to discover the full spectrum of AD genes across the whole genome.

**Results:**

We classified AD candidate genes with an accuracy and the area under the receiver operating characteristic (ROC) curve of 84.56% and 94%. Our approach provides a supplement for the spectrum of AD-associated genes extracted from more than 20,000 genes in a genome wide scale.

**Conclusions:**

In this study, we have elucidated the whole-genome spectrum of AD, using a machine learning approach. Through this method, we expect for the candidate gene catalogue to provide a more comprehensive annotation of AD for researchers.

**Electronic supplementary material:**

The online version of this article (10.1186/s12883-017-1010-3) contains supplementary material, which is available to authorized users.

## Background

Alzheimer’s disease (AD) is a widespread progressive neurodegenerative disease type, characterized by impaired memory, cognitive functioning, and changed behavior [1]. Past genetic research implicates b-amyloid peptide accumulation and deposition, as well as tau protein pathology, selective neuronal death, synaptic and neurotransmitter loss, and neuroinflammation in Alzheimer’s disease pathogenesis [2]. However, the standard of research into AD is Genome Wide Association Studies (GWAS) with pedigree analysis, rather than candidate pathway exploration. Therefore, the understanding of AD is limited by sample size and quality, making it a challenge to have overall insight into AD. Moreover, AD heritability is estimated at ~60–80% [3], while the genetic architecture of AD is imperfectly characterized.

Complex human diseases such as AD are caused by the composite action of multiple, disease-related genes. At present, AD has at least 4 well-known disease-causing genes: the amyloid precursor protein (*APP*) gene and the Presenilin (*PSEN1*/*PSEN2*) genes for familial AD, and apolipoprotein E (*APOE*) ε4 for sporadic AD [1]. A key goal of biomedical research is to seek out disease risk genes, and to elucidate the function of these risk genes in the development of disease and the complex networks of gene-gene interactions underlying complex traits [4]. For this purpose, expanding the AD-associated gene set is necessary. However, with the rapid development of sequencing technology, large amounts of new sequence data must be analyzed to extract disease-related genes using novel, computational approaches.

Thus far, methods based on different data-types and different strategies have been applied in predicting AD-associated genes. Prediction methods can be roughly divided into five types: methods integrating protein-protein interaction networks with information such as protein subcellular localization, gene expression quantification, or gene functional annotation [5–8]; patterns of sequence-based features shared by disease genes [9–11]; machine learning and network topological features [12]; or information about tissue-specific networks [13, 14]. In past research, these methods have been applied to predict associated genes or biomarkers [15–17]. But there are few reports on the predictions based on the brain gene expression data.

We here present a genome-wide method using human brain-specific gene interaction network constructed by gene expression data, resulting in predictions for AD candidate genes. The brain-specific network works by integrating relations between each pair of AD-associated genes, in order to present how genes function together in the brain. This disease-gene classifier extracts the correlation coefficients of known AD-associated/AD-unassociated genes in this brain network, and then uses the coefficients specific to AD-associated/AD-unassociated genes to predict the level of potential AD association for every gene in the genome. After this initial prediction, we then select the predicted AD-related genes with GO functional annotations which are same as those of most of known AD-association genes as the candidate risk genes or biomarkers for AD. In addition, we compared the sequence-based features of all genes, in order to assess our approach. Furthermore, we found that some of our predictions are consistent with associations reported elsewhere in the AD literature, validating the result. The genome-wide complement of Alzheimer’s candidate genes predicted in this study can thus be used to explore the mechanism of AD, and ultimately, to assist in the discovery of better treatments for AD.

## Methods

### Data sources

#### AD related genes

We collected 335 AD-associated genes from public Alzheimer’s disease databases (AlzGene, http://www.alzgene.org/) and from publications treating upon AD. Then, we collected total 22,646 genes and removed the 335 AD-associated genes and genes recorded in OMIM (https://www.omim.org/) as our initial control dataset. Finally, we selected 335 AD non-associated genes (the same number of AD-associated genes) from the initial control dataset with the minimal interaction between 335 AD-associated genes (*Optimal Control Dataset*). At the same time, we randomly selected the other dataset of 335 non-associated genes (*n* = 100) for SVM training, but the *Optimal Control Dataset* had the highest correct rate (Additional file 1: Figure S1).

#### Gene-gene interaction data

The machine learning method used require a set of known gene-gene interaction data for the model input. We obtained this data from GIANT [18] (http://giant.princeton.edu), which can be set to extract the subset of tissue-specific interactions. Since the pathogenesis of Alzheimer’s disease is associated with brain tissue, we selected brain-specific, functional gene interaction data.

### Prediction

There are many different supervised machine learning approaches. We implement a prediction algorithm by SVM (Support Vector Machines) using the e1071 package of R, published by David Meyer. SVM was chosen here, as it can allow the assignment of different weights to different classes. The kernel type used in training and predicting is radial. In addition, a 5-fold cross validation was performed, in order to assess the quality of the model.

## Results

**Fig. 1:**
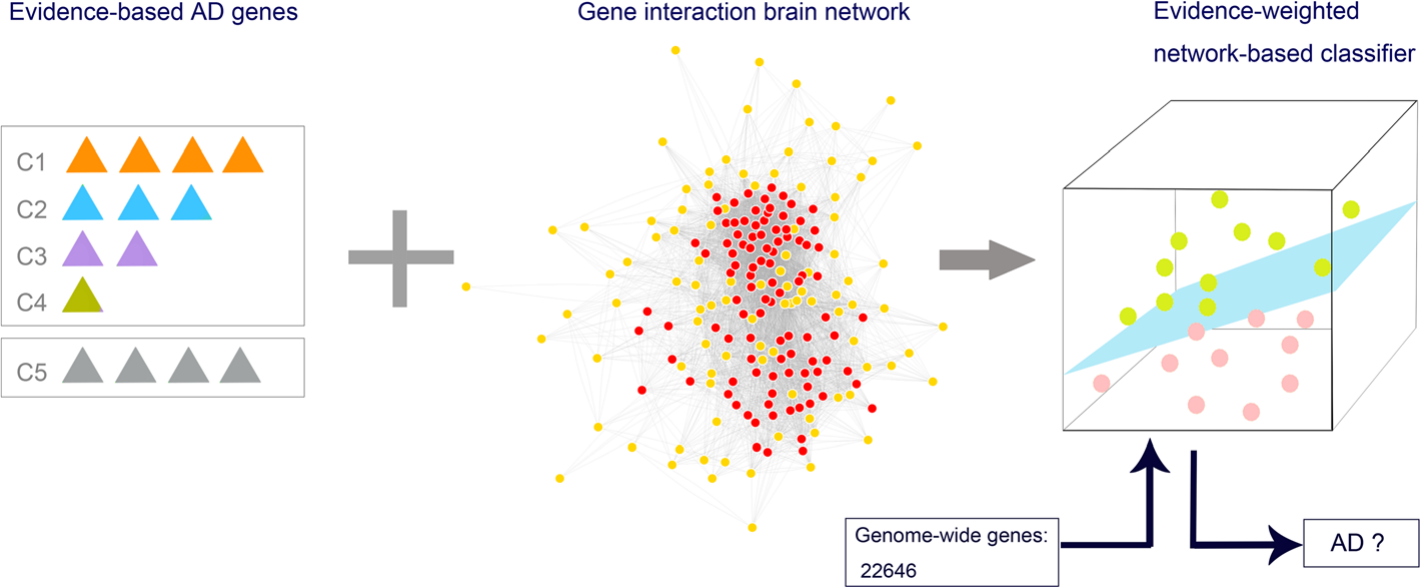
Genome-wide prediction of Alzheimer-associated genes. Our prediction was based on machine learning methods that are trained upon the already associated AD genes, which are already linked to AD at various levels of evidence (C1AD-C4AD), as a positive training set, and other genes excluded from the OMIM database (C5) as a negative training set. Combining these with the human brain-specific gene network, we were able to build an evidence-weighted, network-based classifier, and predict the probability of the association between each gene and AD across the genome

We used human, tissue-specific networking to discover the full spectrum of AD genes across the whole genome. We then assessed the reliability of these genes (Fig. 1). Here, we depict the AD genetic spectrum, and provide an overview of the AD associated genes, honing the focus of further AD study for future researchers.

### Alzheimer’s disease genes spectrum

After initially collecting 335 AD-associated genes [Additional file 2], we then classified these genes into four categories, based on the strength of supporting evidence (the number of positive evidence of family-based studies and case-control studies). These were labeled C1-AD: probable pathogenic genes. C2-AD: high confidence genes. C3-AD: related genes, and C4-AD: possibly associated genes. Three hundred thirty-five AD-non associated genes as C5 group [Additional file 3]. Our goal is, within the scope of whole genome analysis, to find a stable relationship between a pair of genes, so as to find the AD candidate genes closely linked to genes known to be associated to AD.

**Fig. 2:**
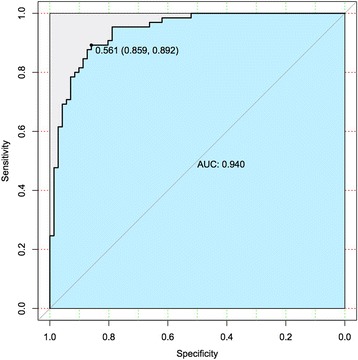
Receiver operating characteristic (ROC) curve for SVM model classification effect. The threshold for the ROC was 0.561. At this threshold, sensitivity was 0.859, specificity was 0.892, area under the curve (AUC) was 0.94

According to the pathogenic mechanism of AD, we recruited the brain-specific gene network data from GIANT. Using these five gene groups, along with their evidence classification, and integrating brain-specific gene network data, we trained an evidence-weighted, network-based classifier, using an SVM approach. We randomly subdivided the total genes into two parts (10-fold cross-validation), which were used as training dataset and testing dataset, respectively. The classifier first identified network patterns (The relationship between any gene and 670 genes (335 AD associated genes +335 AD non-associated genes) as the features of SVM model). Then, we used the testing dataset to test the accuracy of the classifier, and divided them into initial two categories (AD related and AD non-related). We found that the average correct rate was 80.59% and the highest correct rate reached 84.56% with the ROC curve (receiver operating characteristic curve) as shown in Fig. 2. The ROC curve was constructed by “*ROCR*” package in R with a threshold of 0.561. Finally, we applied this classifier integrating the classification of known AD-associated genes to identify new AD candidate genes which interact closely with the known AD associated genes in the brain-specific network and divided those candidate genes into different groups by comparing the probability of each group and choosing the largest one. This method resulted in a comprehensive, genome-wide, ranked list of AD candidate genes.

To screen a more credible candidate gene list, we first annotated all AD associated genes (known AD-associated genes and AD predicted candidates) using the Gene Ontology resource (GO: http://www.geneontology.org/). We then performed a GO functional enrichment analysis of 335 known AD-associated genes and selected the top 10 GO items (Table 1) with *P*-values (adjusted by false discovery rate [19]) below a max value of 6.87e-11. Finally, we chose the AD predicted genes annotated on those GO items to further obtain high-confidence candidate genes. After this filter, we arrived at a total number of 832 AD predicted genes [Additional file 4].

**Table 1:** Top ten GO items of significantly enriched AD-associated genes

Cluster	*P*-values (FDR)	Information
GO:0005515	5.70e-19	Protein binding
GO:0005615	5.34e-15	Extracellular space
GO:0042493	6.59e-15	Response to drug
GO:0042157	9.57e-15	Lipoprotein metabolic process
GO:0008203	1.68e-14	Cholesterol metabolic process
GO:0009986	5.34e-13	Cell surface
GO:0042802	1.61e-12	Identical protein binding
GO:0019899	3.24e-12	Enzyme binding
GO:0044281	3.55e-11	Small molecule metabolic process
GO:0001540	6.87e-11	Beta-amyloid binding

### The assessment of the full AD genes spectrum

In previous research, one prediction method has been based upon similarities in sequence-based features in disease genes [9–11]. Herein, a set of features was chosen from the genes, in order to assess our predictions.

The feature set (described in Table 2) reflects the structure and content of each gene examined. Table 3 lists the differences among the features present among the different sets of genes. Using the Mann-Whitney U test, we discovered highly significant differences in gene length, exon count, transcript count, 3’UTR length and 5’UTR length between the gene sequences of the AD-associated gene set and the control set of genes. Besides, there were also highly significant differences in transmembrane domain, signal domain and paralog calculated using the chi squared test. We reached the same conclusion when comparing the AD candidate dataset and control dataset.

**Table 2:** The list of selected features of gene sets for comparison

Feature	Source	Description
Gene length	Ensembl [29]	Length of gene in bp
Protein length	UniProt [30]	Length of protein in aa
CDS length	Ensembl	Length of coding sequence in bp
Length of 3’ UTR	Ensembl	Length of the 3′ untranslated region in bp
Length of 5’ UTR	Ensembl	Length of the 5′ untranslated region in bp
Transcript count	Ensembl	Transcript count in the gene
Number of exons	Ensembl	Number of exons in the gene
GC content	Ensembl	GC content (%) of gene
Transmembrane domain	Ensembl	If the gene has a transmembrane domain
Signal domain	Ensembl	If the gene has a signal domain
Paralog	Ensembl	If the gene has a paralog in the human genome

**Table 3:** Significant differences among the AD-associated set, control set and predicted AD candidate set

Feature	AD-related dataset (median)	Control dataset (median)	AD-predicted dataset (median)
Gene length (bp)	43,474.5	8906	36,937
Length of 3’ UTR (bp)	309	103	362
Length of 5’ UTR (bp)	345	134	332
Transcript count	8	3	8
Number of exons	10	5	10
Transmembrane domain	31.31%	23.18%	32.04%
Signal domain	33.43%	14.09%	33.86%
Paralog	81.79%	45.97%	86.21%

Meanwhile, there was no statistically significant difference between the sequence patterns of AD-associated genes and AD candidate genes (as described in Table 4). The size (in bp) of the genes in both the AD-associated dataset and the AD candidate dataset is significantly larger than among controls, and this is similar to previous findings on AD association [9], which also report generally that genes associated with disease tend to be larger than those involved in normal phenotypes. In much the same way as larger total gene length is associated with AD, the length of 3’ UTR and 5’UTR, transcript count and the number of exons per gene, is in the AD-associated dataset and AD candidate dataset all larger. Genes in the known AD-associated dataset and the AD candidate dataset had a median count of 10 exons and 8 transcripts, while genes in the control dataset had a median count of 5 exons and 3 transcripts. We also found that there were significant differences in the length of 3’ UTR and 5’UTR in both AD-associated genes and AD candidate genes, which both had a larger median length of 3’UTR and 5’UTR, while genes in the control set had a smaller median length (described in Table 3).

**Table 4:** The differences between any two of the four datasets calculated by the *P* value of Mann-Whitney U test or Chi-squared test

Features		AD-associated dataset	Control dataset	AD-predicted dataset	Non-mental-health dataset
Gene length (Mann-Whitney U test)	AD- associated set	–	< 2.2E-16	0.01607	0.002573
	Control dataset	< 2.2E-16	–	< 2.2E-16	< 2.2E-16
	AD-predicted dataset	0.01607	< 2.2E-16	–	0.2018
	Non-mental-health dataset	0.002573	< 2.2E-16	0.2018	–
Length of 3’ UTR (Mann-Whitney U test)	AD-associated set	–	< 2.2E-16	0.109	0.1131
	Control dataset	< 2.2E-16	–	< 2.2E-16	< 2.2E-16
	AD-predicted dataset	0.109	< 2.2E-16	–	0.0003546
	Non-mental-health dataset	0.1131	< 2.2E-16	0.0003546	–
Length of 5’ UTR (Mann-Whitney U test)	AD-associated dataset	–	1.17E-13	0.4351	0.008426
	Control dataset	1.17E-13	–	< 2.2E-16	3.05E-13
	AD-predicted dataset	0.4351	< 2.2E-16	–	0.000159
	Non-mental-health dataset	0.008426	3.05E-13	0.000159	–
Transcript count (Mann-Whitney U test)	AD-associated dataset	–	< 2.2E-16	0.2962	0.0006213
	Control dataset	< 2.2E-16	–	< 2.2E-16	< 2.2E-16
	AD-predicted dataset	0.2962	< 2.2E-16	–	0.0006213
	Non-mental-health dataset	0.0006213	< 2.2E-16	0.0006213	–
Number of exon (Mann-Whitney U test)	AD-associated dataset	–	< 2.2E-16	0.1314	0.3506
	Control dataset	< 2.2E-16	–	< 2.2E-16	< 2.2E-16
	AD-predicted dataset	0.1314	< 2.2E-16	–	0.1537
	Non-mental-health dataset	0.3506	< 2.2E-16	0.1537	–
Transmembrane domain (Chi-square test)	AD- associated set	–	0.03783	0.8109	0.6143
	Control dataset	0.03783	–	0.01107	0.0448
	AD-predicted dataset	0.8109	0.01107	–	0.302
	Non-mental-health dataset	0.6143	0.0448	0.302	–
Signal domain (Chi-square test)	AD- associated set	–	3.70E-07	0.89	3.54E-05
	Control dataset	3.70E-07	–	1.20E-08	0.006176
	AD-predicted dataset	0.89	1.20E-08	–	1.12E-08
	Non-mental-health dataset	3.54E-05	0.006176	1.12E-08	–
Paralog (Chi-square test)	AD- associated set	–	< 2.2E-16	0.05604	0.0007944
	Control dataset	< 2.2E-16	–	< 2.2E-16	< 2.2E-16
	AD-predicted dataset	0.05604	< 2.2E-16	–	5.19E-13
	Non-mental-health dataset	0.0007944	< 2.2E-16	5.19E-13	–

Furtherly, we added genes annotated to non-mental-health diseases for comparison [14]. Differences calculated as *p*-value by using the Mann-Whitney U test or chi squared test between any two sets of four gene sets are shown in Table 4. On the basis of above results, we guessed that the differences between the sequence patterns of AD-associated genes and non-mental-health genes (as Group 1) were greater than that of AD-associated genes and AD candidate genes (as Group 2), but less than that of AD-associated genes and control genes (as Group 3). That is to say, our guess was that the *p*-values of Group 1 were smaller than that of Group 2, but larger than that of Group 3. Finally, most of the results were up to our expection, including length of gene and 5’ UTR, transcript count, transmembrane domain, signal domain and paralog.

**Fig. 3:**
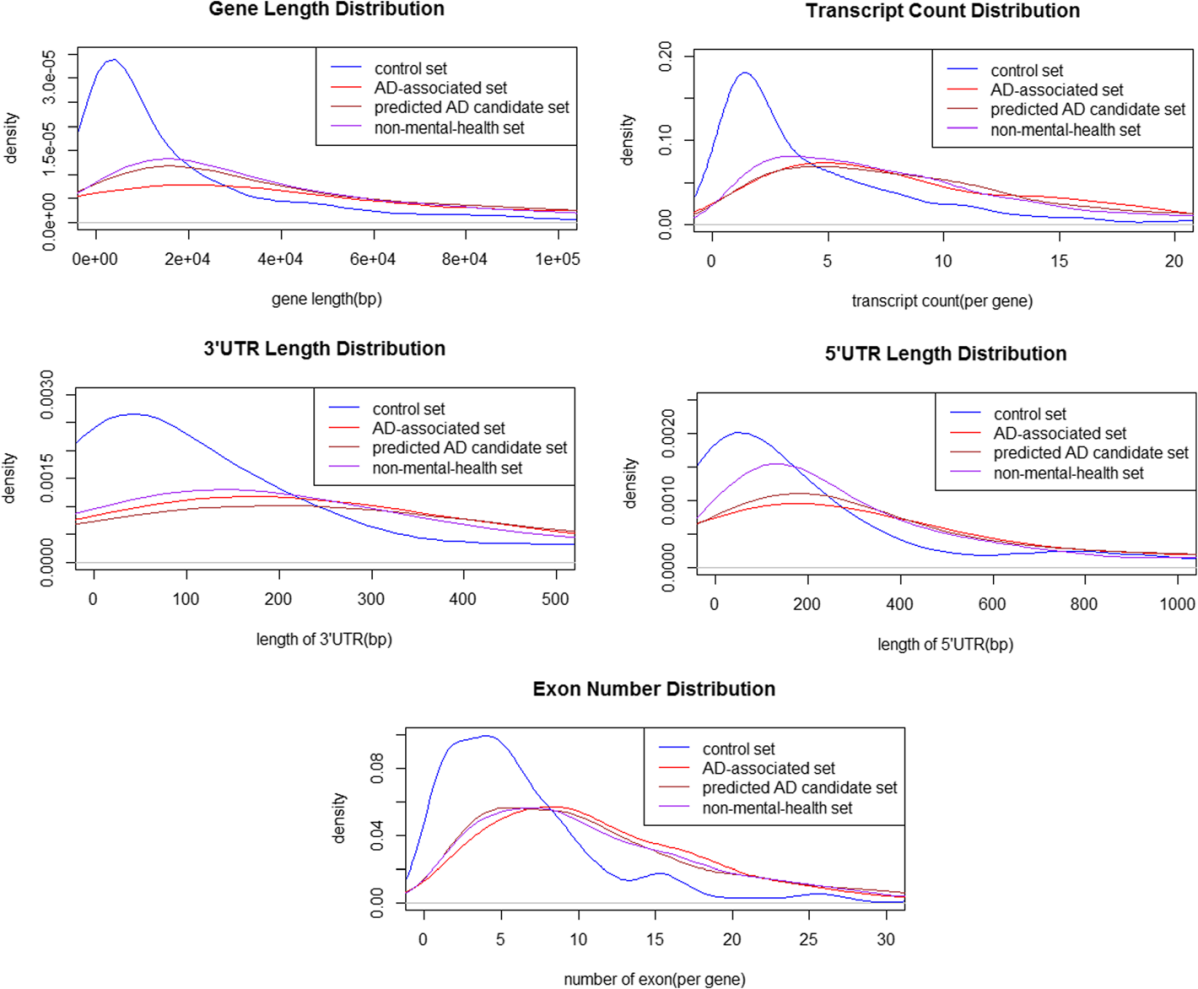
Distributions of selected features of different dataset. Distributions of predicted AD candidate set are basically consistent with those of AD-associated set; rather, distributions of control set are quite different from those of AD-associated set and predicted AD candidate set

Graphs presenting the distributions of each feature in the four datasets are shown in Fig. 3. It’s clear that the peaks of feature values of the known AD-associated dataset and AD candidate sets shift rightward, when compared to those of the control set. To our knowledge, the number of exons is also correlated to total gene length. However, the differences in 5’ UTR and 3’ UTR length have not been explained in terms of correlations to other feature differences.

Since the genes of the known AD-associated dataset were selected from the literature published before 2015, we also checked the predicted AD candidate gene set by scanning papers published post-2014 to verify the accuracy of our prediction (described in Table 5). As a result, we found that the AD candidate genes which were also reported in the post-2014 research could be roughly divided into several types. First, our gene candidates were identified as being associated with AD genes [20–22]. For example, DAB1, a novel candidate liability/protective gene, was identified by functional enrichment analysis of 3 AD Genome-Wide Association Studies (GWAS). Second, the genes were simply associated with risk of AD [23–25]. ANXA1 and CDC25C were identified as potentially contributing to AD susceptibility, by applying ICSNPathway analysis to the AD GWAS meta-analysis data. Third, abnormal changes in gene modification level or expression level were shown to exist in AD cases, when compared to the controls [23, 25]. For instance, DNA methylation levels within the CRTC1 gene were decreased in human hippocampus tissue affected by AD, suggesting that CRTC1 methylation plays an important role in AD pathophysiology.

**Table 5:** Information about discovering AD-associated genes from published papers since 2015

Articles	Total genes	Trained genes	Predicted genes
Chen J A, et al. [22]	DYSF, PAXIP1	–	PAXIP1
Xiao Q, et al.[31]	CD2AP,SORL1, FERMT2,PVRL2, TOMM40	SORL1, FERMT2, TOMM40	PVRL2
Gao H, et al. [20]	DAB1	–	DAB1
Malishkavich A, et al. [32]	ADNP	–	ADNP
Lee Y H, et al. [23]	ANXA1, CDC25C	–	ANXA1, CDC25C
Zheng X, et al. [33]	APC2	–	APC2
Lin Q, et al. [24]	APOA1,APOC3, APOA4	APOA1, APOA4	APOC3
Marchesi V T, et al. [34]	NLRP3,APP, TREX1,NOTCH3, COL4A1	APP	NLRP3, COL4A1
Total	20	6	10

## Discussion

In this study, we have elucidated the whole-genome spectrum of AD, using a machine learning approach. We have classified the collected AD-associated genes by potential pathogenesis and taken advantage of brain-specific function networking to obtain correlations within the activity of any given pair of genes. Through this method, we expect for the candidate gene catalogue to provide a more comprehensive annotation of AD for researchers. Furthermore, this method could be applied to other brain disease pathogenic genes prediction, such as Parkinson’s disease, schizophrenia and so on.

By comparing the AD gene dataset with the control gene dataset in the sequence-based features (Tables 3 and 4), we found the median length of AD genes was much longer than controls. We speculate that the longer the gene is, the more mutations it will accumulate and the greater the pathogenicity it will be. Therefore, we suggest the research of related disease genes could be from the perspective of gene mutation load. What’s more, the proportion of genes with paralogs in the AD dataset is greater than in controls. It has been found human monogenic disease genes have frequently functionally redundant paralogs [26], but only one of the paralogous gene is associated with disease [27]. AD as a typical complex disease driven by multiple factors, the role of paralogs of pathogenic genes is worth further investigation.

The human brain has enormously complex cellular diversity, different parts of the neuron with different gene expression are specialized for different functions [28]. And AD is not caused by the role of single gene, so we need from the global point of view to study its development mechanism. The AD gene dataset obtained in our study can be used to explore the differences in gene expression and rare mutations distribution between AD patients and normal controls in different brain region, and clinically analyze the overexpression in AD brain neurons by single cell sequencing.

According to the GO functional enrichment analysis, we found AD-associated genes may play a role in the following GO items: protein binding, extracellular space, drug response, lipoprotein metabolic process, cholesterol metabolic process, cell surface, enzyme binding, beta-amyloid binding. To our best knowledge, most of them were concerned by different researchers, but there were no reports on drug response and enzyme binding, which may be a direction of our future study.

## Conclusions

In this study, we have elucidated the whole-genome spectrum of AD, using a machine learning approach. Through this method, we expect for the candidate gene catalogue to provide a more comprehensive annotation of AD for researchers.

## Additional files

Additional file 1: Figure S1. The correct rates of different non-associated gene sets in SVM training. We randomly selected the dataset of 335 non-associated genes (*n* = 100) for SVM training. (TIFF 12473 kb)

Additional file 2: 335 AD-associated genes. The datasets collected from public Alzheimer’s disease databases (AlzGene) and the publications treating upon AD. (XLSX 14 kb)

Additional file 3: The classification of AD-associated genes and AD-non associated genes. C1-AD: probable pathogenic genes; C2-AD: high confidence genes; C3-AD: related genes; C4-AD: possibly associated genes; C5-AD: AD-non associated genes. (XLSX 19 kb)

Additional file 4: 832 AD predicted genes. A total number of AD predicted genes across the whole genome in our study. (XLSX 68 kb)
