# Supplementary figures and images for: Revealing Alzheimer’s disease genes spectrum in the whole-genome by machine learning
Source: BMC Neurol. 2018 Jan 10;18:5. doi: 10.1186/s12883-017-1010-3 (PMC5763548; doi:10.1186/s12883-017-1010-3)

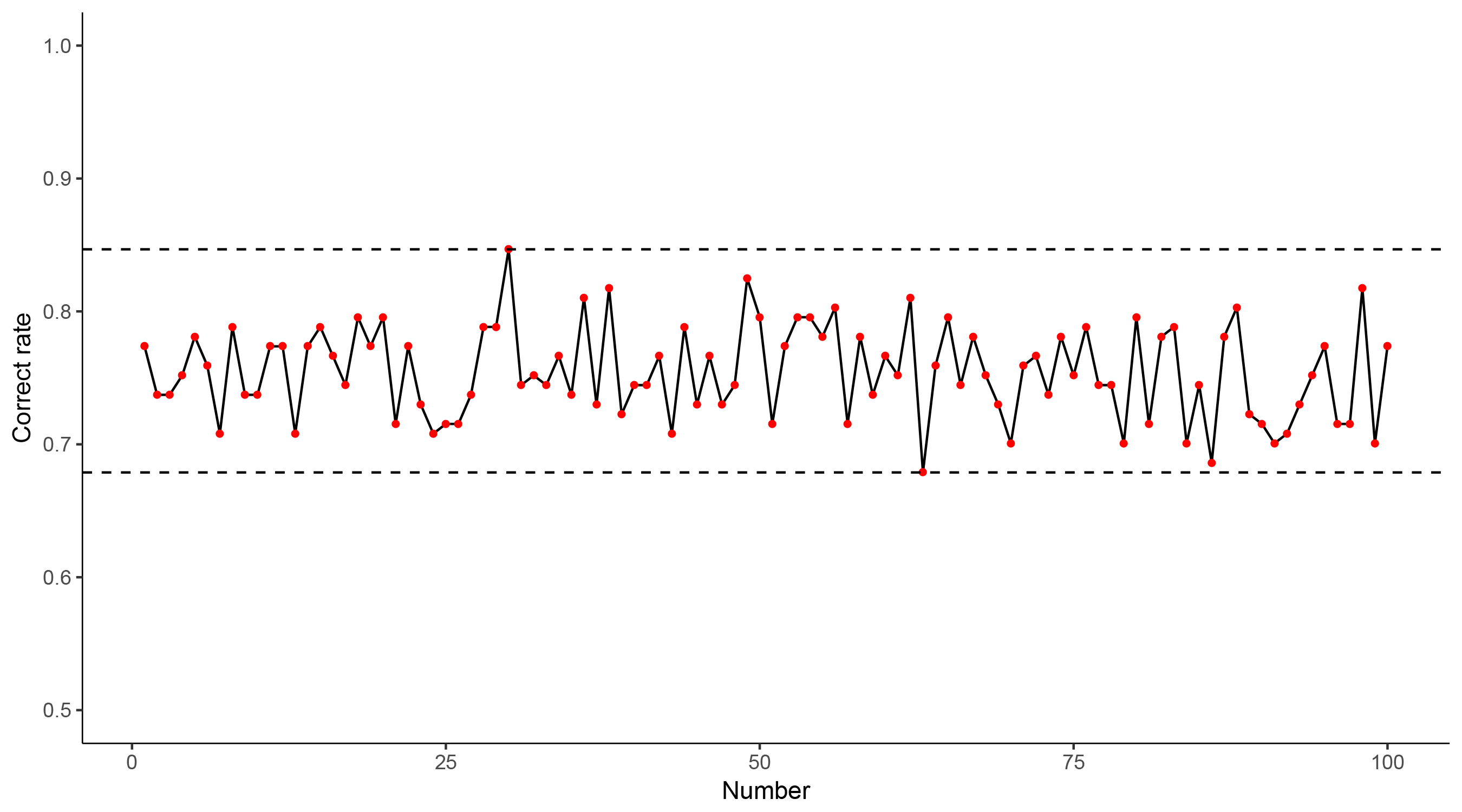

Supplement: Supplementary file 1 — The correct rates of different non-associated gene sets in SVM training. We randomly selected the dataset of 335 non-associated genes (n = 100) for SVM training. (TIFF 12473 kb) [file 12883_2017_1010_MOESM1_ESM.tif]
